# The oral microflora in obesity and type-2 diabetes

**DOI:** 10.3402/jom.v4i0.19013

**Published:** 2012-10-30

**Authors:** Edward Shillitoe, Ruth Weinstock, Taewan Kim, Howard Simon, Jessica Planer, Susan Noonan, Robert Cooney

**Affiliations:** Department of Microbiology and Immunology, SUNY Upstate Medical University, Syracuse, NY, USA

**Keywords:** oral microflora, type-2 diabetes, obesity, gastric bypass, *Bifidobacteria*

## Abstract

**Background:**

Type 2 diabetes mellitus (T2DM) is prevalent in people with obesity. It has been proposed that these conditions are related to specific features of the microflora of the mouth and lower gastrointestinal (GI) tract. Hyperglycemia often resolves quickly after Roux-en-Y gastric bypass (RYGB) but the role of the GI microflora cannot be examined easily because of reduced intestinal mobility. We propose that the study of microorganisms present in the mouth of patients undergoing RYGB will contribute to our understanding of the role of bacteria in the pathogenesis of T2DM.

**Objective:**

To conduct a feasibility study to examine differences in oral microbes in obese patients with and without T2DM and to determine whether it is feasible to measure changes after gastric bypass surgery.

**Methods:**

Individuals with morbid obesity (*n*=29), of whom 13 had T2DM, were studied. Oral rinses, stool samples, and blood samples were obtained before RYGB, and oral rinses and blood samples were obtained at 2 and 12 weeks postsurgery.

**Results:**

Prior to surgery, participants with T2DM had slightly higher total levels of oral bacteria than those without diabetes. Those with HbA1c > 6.5% had rather lower levels of *Bifidobacteria* in the mouth and stool. At 2 weeks post-RYGB, patients with T2DM were able to reduce or discontinue their hypoglycemic medications. Stool samples could not be obtained but oral rinses were readily available. The levels of oral *Bifidobacteria* had increased tenfold and levels of circulating endotoxin and tumor necrosis factor-alpha had decreased.

**Conclusions:**

The study of oral bacteria before and after RYGB is feasible and should be tested in larger patient populations to increase our understanding of the role of microorganisms in the pathogenesis of obesity and T2DM.

Obesity is increasing in prevalence and has now reached epidemic proportions in the United States (1). Obesity is associated with type 2 diabetes mellitus (T2DM), periodontal disease, and other comorbidities (2). Although the mechanism of these associations is not entirely clear, an increasing body of evidence implicates the microbiota of the gastrointestinal (GI) tract.

Obesity and T2DM can be induced in mice by a high fat diet, but only if the mice have a normal GI microflora (3). If antibiotics change the microflora, weight loss and improved glycemic control are observed (4). These effects are associated with elevated plasma levels of endotoxin (5), which can stimulate elevations in circulating levels of inflammatory cytokines such as tumor necrosis factor-alpha (TNF-α) (6), causing insulin resistance (7). Although no specific GI pathogens have been discovered that induce obesity or diabetes in mice, the conditions are associated with reduced abundance of *Bifidobacteria* (8, 9).

Human obesity and T2DM show several similarities to the animal model. Obese individuals have differences in their GI microflora from non-obese individuals and the flora changes when weight is lost (10). The oral cavity of obese individuals has higher levels of various bacteria (11–13), whereas patients with T2DM have fewer *Bifidobacteria* in the intestine than control subjects (14). In addition to the bacterial features, obesity, T2DM, and periodontal disease are associated with elevated circulating levels of inflammatory markers such as endotoxin and TNF-α (15, 16).

Although the clinical association between obesity and its comorbidities is consistent with the changes in the microflora, the relevant mechanisms remain obscure. The interpretation of data is complicated by the fact that patients gain and lose weight slowly. Consequently, changes in adiposity, glycemic control, the microbial flora, and the inflammatory markers all occur within the same time frame. One approach to separating these effects is to study individuals who are undergoing Roux-en-Y gastric bypass surgery (RYGB), which results in improved glycemic control within days, before significant weight loss has occurred (17). The reasons for this sudden improvement are unknown and also the role of microorganisms has not been assessed because there is a postsurgical loss of intestinal mobility, which prevents the collection of stool samples. The current study investigated the feasibility of studying differences in oral microflora in obesity with and without T2DM.

## Methods

Consecutive patients with morbid obesity who had been selected to undergo RYGB at SUNY Upstate Medical University between August and December 2010 were invited to participate. All participants met the criteria for bariatric surgery established by the NIH Consensus Conference (18). Exclusion criteria included current smokers and those having received antibiotics within the previous 6 months. The project was approved by the Institutional Review Board for the Protection of Human Subjects and all participants provided signed informed consent.

Prior to surgery, the height and weight of each subject was measured, and each maintained a diet journal in which they recorded all food and drink for 3 consecutive days. An oral examination was performed by the principal investigator to determine the presence of periodontal pockets of 4 mm depth or greater. Before surgery all subjects provided an oral sample by rinsing their mouths for 30 seconds with 10 ml of ice-cold saline and provided a fresh stool sample. A non-fasting blood sample was obtained, the level of HbA1c was determined, and the serum was separated. Oral, stool, and serum samples were immediately frozen and stored at −80°. All participants were assigned study identifier numbers and the staff members performing analyses were blind to group allocation.

On the day of surgery, each subject was given a single prophylactic intravenous dose of cephazolin. Clindamycin was administered to penicillin-allergic patients, as per usual surgical practice. No other antibiotics were given to any subject at any time during the study. The surgical procedure consisted of open or laparoscopic RYGB as previously described (19).

At 2- and 12-weeks after surgery, oral rinses and blood samples were obtained as before, and the subjects’ weight was recorded. Again, the subjects provided a diet journal. Those subjects with T2DM continued to monitor their blood glucose levels, and their glucose-lowering medications were reduced or stopped as indicated by their physician.

### Markers of inflammation

Serum levels of endotoxin were determined with a Limulus Amebocyte Lysate assay (Associates of Cape Cod Inc., Falmouth, MA). Serum levels of TNF-α were measured with a Quantikine ELISA assay (R&D Systems, Minneapolis MN). Serum levels of CRP were determined using the high-sensitivity C-reactive protein ELISA assay (Calbiotech Inc., Spring Valley, CA).

### Microflora

DNA was isolated from 1.5 ml of each oral rinse using the DNeasy Blood and Tissue kit (Qiagen Inc, Valencia, CA) and from 0.2 g of each stool sample using the QIAmp DNA stool kit (Qiagen). A real-time quantitative PCR (RTq-PCR) was used to determine the concentration of ‘All Bacteria’, *Firmicutes*, *Bacteroidites*, *Bifidobacteria*, *Bacteroides thetaiotaomicron*, *Porphyromonas gingivalis* and *Methanobrevibacter smithii*, using previously published primer pairs (20–25). The RTq-PCR was calibrated with DNA from cultures of reference strains.

### Statistical analysis

Data are expressed as the mean±SEM for each group. Differences between groups were assessed by student's *t*-test, using the computer program Prism (GraphPad Software Inc., La Jolla, CA). A *p*-value < 0.05 was taken as indicator of statistically significant difference. To find what sample sizes would be needed to evaluate likely differences between patient groups, a power analysis (26) was performed.

## Results

### Clinical data

Participants (*n*=29) with morbid obesity were enrolled consecutively over a period of 4 months; 7 males, 22 females, mean age 41 years (range 23–55), and mean body mass index (BMI) 48 (range 37–97). Prior to surgery, 13 participants had been diagnosed as having T2DM by their primary care physician, using standard criteria. All 13 were receiving oral hypoglycemic medications for T2DM, and 3 were also taking insulin. The HbA1c levels of all participants ranged from 5.2% to 11.3% with 8/13 with T2DM having a level > 6.5%.

One subject was edentulous and no oral rinses were obtained from this patient. All other patients had 8 to 30 teeth (mean 24.4±0.9). Two patients had a single periodontal pocket of 4 mm depth and the others had no pockets. None of the patients therefore met any of the current definitions of periodontitis (27).

**Fig. 1:**
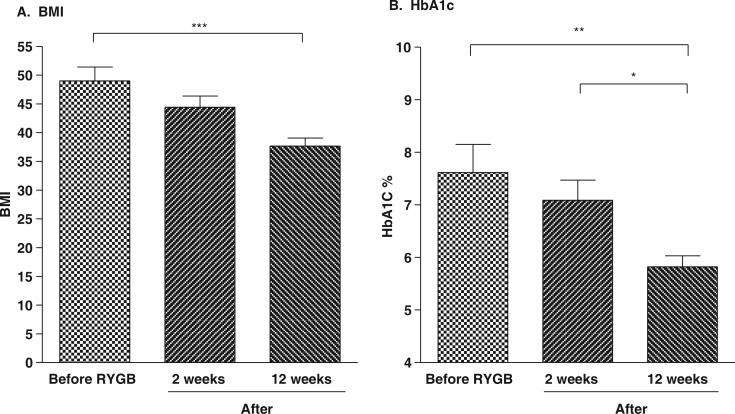
BMI and HbA1c. Effect of RYGB on BMI in all subjects (A) and on HbA1c levels in subjects with T2DM (B). *p<0.05, **p<0.01, ***p<0.001.

Immediately after surgery, medications for the treatment of diabetes were completely discontinued for 11 of the 13 subjects with diabetes and dosages were reduced for the remaining 2. All post-RYGB subjects with T2DM noted that their blood glucose levels were maintained below 200 mg/dL and the improvement in glycemic control was confirmed by the subsequent reduction in HbA1c levels (Fig. 1B). At 14 days postsurgery, dietary intake of carbohydrates, protein, and fat were all significantly reduced (*p*<0.0001). Post-RYGB weight loss during the first 2 weeks after surgery was not significant (*p*>0.05) and all subjects remained morbidly obese. However, by 12 weeks post-RYGB, the weight loss was significant (*p*<0.001, Fig. 1A).

### Markers of inflammation

**Fig. 2:**
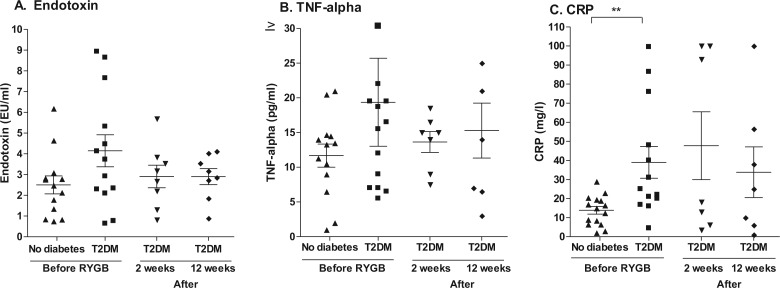
Inflammatory markers. Serum levels of endotoxin (A), TNF-α (B), and CRP (C) in subjects with or without T2DM before RYGB and levels in subjects with T2DM at 2 weeks and 12 weeks after surgery. **p<0.01.

Prior to surgery, there was a higher baseline serum endotoxin level in subjects with T2DM (4.15±0.77 EU/ml) than in those without diabetes (2.51±0.43 EU/ml). Two weeks after surgery, endotoxin levels in subjects with T2DM had fallen to 2.91 + 0.54 EU/ml and remained at that low level up to week 12 (Fig. 2A).

Prior to surgery, the serum levels of TNF-α were also higher in the participants with T2DM (19.36±6.34 pg/ml) than in those without diabetes (11.68±1.67 pg/ml). At 2 weeks after surgery, levels in the subjects with T2DM had fallen to 13.64±1.50 pg/ml and remained at that level at week 12 (Fig. 2B).

Serum levels of CRP, prior to surgery, were significantly higher in the patients with T2DM (39.0±8.3 mg/l) than in those without diabetes (13.8±2.0 mg/l, *p*<0.01). The mean level in the subjects with T2DM was slightly increased after surgery (Fig. 2C).

### Microflora

The RTq-PCR was found to be highly specific. Each primer pair induced amplification of the appropriate reference DNA only, except that the bacteroidites primer amplified DNA from *P. gingivalis* and *B. thetaiotaomicron* with almost equal efficiency, consistent with their known relatedness (28). The ‘All Bacteria’ primers did amplify DNA of *M. smithii*, which is consistent with its original classification as a bacterium.

**Fig. 3:**
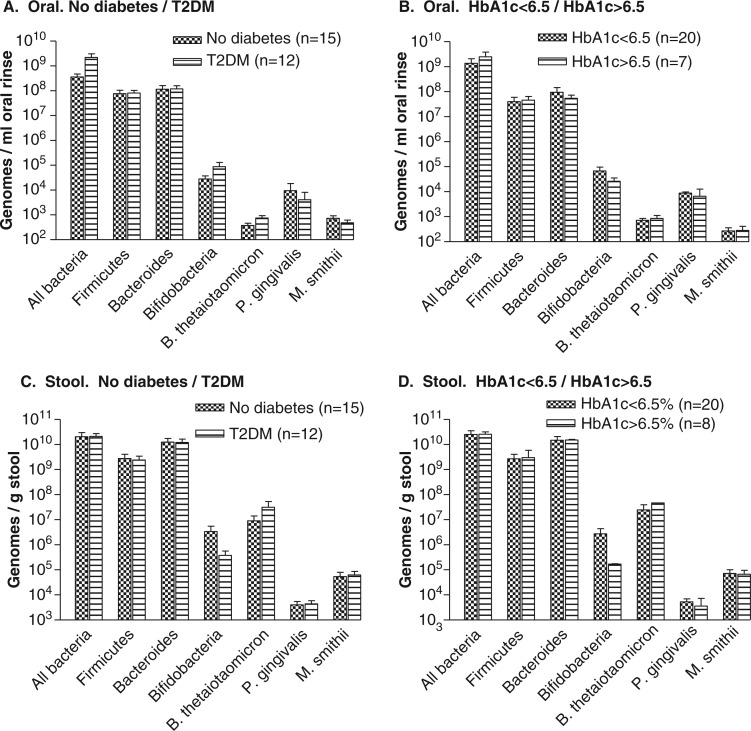
Pre-surgical microflora. Concentrations of microbes in oral rinses (A, B) and stool (C, D) before RYGB, with subjects stratified as no diabetes/T2DM (A, C) and as HbA1c < 6.5%/HbA1c > 6.5% (B, D).

Prior to surgery, subjects with T2DM had a 10-fold greater concentration of ‘All Bacteria’ in the oral rinses than the subjects without diabetes (2.2×10^9^±8.7×10^8^ genomes/ml compared to 3.6×10^8^±1.17×10^8^) (Fig. 3A). However, when the T2DM subjects were categorized by level of glycemic control, the oral levels of *Bifidobacteria* were lower in the subjects with HbA1c > 6.5% than in those with levels of HbA1c < 6.5% (2.6×10^4^±9.4×10^3^ genomes/ml compared to 6.8×10^4^±2.85×10^4^) (Fig. 3B).

Prior to surgery, there were no differences in stool samples between levels of ‘All Bacteria’ between subjects with or without T2DM (Fig. 2C). However, levels of *Bifidobacteria* were approximately 10-fold lower in subjects with T2DM compared with those without diabetes (3.75×10^5^±1.8×105/genomes/g compared to 3.4×10^6^±2.1×10^6^ genomes/g). This difference was greater when subjects were stratified by HbA1c levels > 6.5% or < 6.5% (1.67×10^5^±4.7×10^3^ genomes/g compared to 2.71×10^6^±1.6×10^6^ genomes/g, Fig. 3D).

**Fig. 4:**
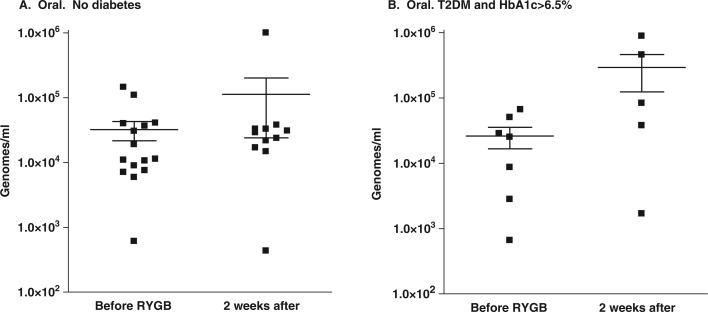
*Bifidobacteria*. Effect of RYGB on oral *Bifidobacteria* of subjects without diabetes (A) or with T2DM and HBA1c > 6.5% (B).

At 2 weeks after RYGB, subjects were not able to provide stool samples reliably because intestinal mobility was still reduced. However, all subjects were able to provide oral rinses. At this time, no organisms showed a change in the oral levels that exceeded 2-fold except for the *Bifidobacteria* species. The oral microflora of the participants without diabetes showed a 2.4-fold increase in the levels of *Bifidobacteria*, from 3.2×10^4^±1.0×10^4^ genomes/ml to 1.1×10^5^±8.9×10^4^ genomes/ml (Fig. 4A). In the participants with T2DM and HbA1c > 6.5%, the level of *Bifidobacteria* in the oral rinses increased approximately 10-fold, from 2.61×10^4^±9.4×10^3^ genomes/ml to 2.92×10^5^±1.69×10^5^ genomes/ml (Fig. 4B). However, all of these changes were at a significance level of *p*>0.05.

### Statistical analysis

Power analysis was applied to find the sample size that would be necessary to detect the differences in the numbers of *Bifidobacteria*, given the means and distributions shown in Fig. 4B. It was determined that to test the hypothesis that the concentrations of *Bifidobacteria* increase after surgery with a power of 80% and α < 0.05, we would require 25 subjects.

## Discussion

The role of microbes in the etiology of T2DM and other comorbidities of obesity might be made clear by studies on patients undergoing RYGB, since glycemic control is reported to improve within days (17). However, this approach is made difficult by the lack of stool samples immediately after surgery, when intestinal mobility is temporarily impaired. Studies at several months after RYGB (29–31) do not reflect immediate (pre-weight loss) postoperative changes. Therefore, in the present study, we examined bacteria from the oral cavity within 2 weeks of surgery when the most important changes are likely to have occurred.

Before RYGB, the subjects with T2DM had slightly higher levels of circulating endotoxin and TNF-α, and significantly higher levels of CRP (Fig. 2), consistent with previous reports (15, 16, 32). At 2 weeks post-RYGB, levels of circulating endotoxin and TNF-α had fallen in subjects with T2DM (Fig. 2). This rapid decline in levels of endotoxin and TNF-α may be related to the changes in the microbiome and thus to the resolution of the hyperglycemia. However, it cannot be excluded that dietary changes were an important factor. The observed changes are not consistent with the notion that an elevated level of TNF-α is due solely to the presence of adipose tissue since the subjects were still morbidly obese. The small increase in the circulating level of CRP after surgery could be a consequence of the surgical procedure itself.

After RYGB, an improvement in glycemic control was seen in subjects with T2DM, with most patients being able to discontinue their hypoglycemic medication. This was expected and is consistent with previous reports (17). The levels of *Bifidobacteria* in stool samples were lower in patients with T2DM prior to surgery, consistent with other reports (14).

Since the clinical and laboratory features of the participants conformed to what might be expected from the previously published literature, the study population does make a suitable group for the study of obesity-related conditions, such as T2DM and periodontal disease. On the contrary, it must be noted that patients undergoing RYGB are not a representative cross section of the global population of obese persons. The availability of RYGB is limited to individuals who meet specific criteria of motivation, compliance and resources. All of the subjects were drawn from the same geographical area, and the sample size was small. Possibly as a result of this selection process, no patient had periodontal disease despite the well-established association of periodontal disease with obesity (2). Therefore, it might be difficult to study the progress of periodontal disease in this group.

The use of oral rinses to evaluate the GI tract microflora was found to be feasible. The major organisms of interest were readily detected in the oral rinses, and changes and differences were consistent with expectations. *Bifidobacteria* in the mouth were somewhat less in the subjects with T2DM who had HbA1c > 6.5% (Fig. 3) and the concentration increased 10-fold within 2 weeks of surgery (Fig. 4). Such large differences were not observed for the other organisms examined. The levels of *Bifidobacteria* in the mouth appeared to reflect those in the lower GI tract, suggesting that a study of oral organisms could provide data with systemic implications. Although the magnitude of the postsurgical change did not achieve the 0.05 level of significance, a power analysis showed that this could feasibly be tested with a larger sample.

Simultaneous changes in oral and lower GI microbes could be due to the surgical procedure correcting a systemic mucosal immune defect as we previously proposed (19, 33). This could allow selected bacterial species to return to normal levels. Alternatively, the oral microflora could directly influence the postgastric microbiota, given that approximately 1 g of bacteria are swallowed per day (34).

Two mechanisms have been proposed to explain the beneficial effects of *Bifidobacteria* and related bacilli in T2DM (8, 9). The first is that they compete with pathogenic organisms, displacing them from the normal microflora (35). If this is the case, then a microbiome-wide study of the oral organisms should identify organisms that are displaced. The second mechanism is that *Bifidobacteria* binds endotoxin to their surface, preventing it from being transported across the mucosal barrier and into the circulation. Although some strains of *Bifidobacteria* are extremely efficient at this, others are not (36). Future studies of the oral microbiome should be aimed at examining variants of *Bifidobacteria* or related organisms and explore possible associations with improvements in glycemic control. Any role for *Bifidobacteria* or related organisms in protection from T2DM or other comorbidities of obesity has obvious implications for contributing to the prevention and treatment of the conditions.

## Conclusion

This study investigated the feasibility of studying the oral microflora in patients undergoing gastric bypass surgery as a novel approach to increasing our understanding of obesity and its comorbidities – T2DM and periodontal disease. The data are consistent with the hypotheses that RYGB affects the microbiome of the GI tract and that studies of the oral flora can provide useful information to direct potential new treatments. Future studies will require larger patient populations, microbiome-wide observations, and investigations of the mechanisms of selected organisms.
